# Electromagnetically-Actuated Reciprocating Pump for High-Flow-Rate Microfluidic Applications

**DOI:** 10.3390/s121013075

**Published:** 2012-09-26

**Authors:** Ming-Tsun Ke, Jian-Hao Zhong, Chia-Yen Lee

**Affiliations:** 1 Department of Energy and Refrigerating Air-Conditioning Engineering, National Taipei University of Technology, Taipei 10608, Taiwan; E-Mail: mtke@ntut.edu.tw; 2 Institute of Materials Engineering, National Pingtung University of Science and Technology, Pingtung 91201, Taiwan; E-Mail: q110023940@yahoo.com.tw; 3 Department of Vehicle Engineering, National Pingtung University of Science and Technology, Pingtung 91201, Taiwan

**Keywords:** electromagnetic pump, electroplating, MEMS, PDMS diaphragm

## Abstract

This study presents an electromagnetically-actuated reciprocating pump for high-flow-rate microfluidic applications. The pump comprises four major components, namely a lower glass plate containing a copper microcoil, a middle PMMA plate incorporating a PDMS diaphragm with a surface-mounted magnet, upper PMMA channel plates, and a ball-type check valve located at the channel inlet. When an AC current is passed through the microcoil, an alternating electromagnetic force is established between the coil and the magnet. The resulting bi-directional deflection of the PDMS diaphragm causes the check-valve to open and close; thereby creating a pumping effect. The experimental results show that a coil input current of 0.4 A generates an electromagnetic force of 47 mN and a diaphragm deflection of 108 μm. Given an actuating voltage of 3 V and a driving frequency of 15 Hz, the flow rate is found to be 13.2 mL/min under zero head pressure conditions.

## 1. Introduction

Rapid advances in micro-electro-mechanical systems (MEMS) techniques over the past few decades have led to the development of many microfluidic devices for use in the chemical, biological and environmental monitoring fields. Typically, these devices are designed to perform specific functions such as sample manipulation, reaction, separation and detection, and so on. Compared to their large-scale counterparts, microfluidic devices have a number of important advantages, including a reduced sample and reagent consumption, a shorter analysis time, and an improved sensitivity. Importantly, two or more of such devices can be integrated on a single chip to construct so-called micro-total-analysis systems (μTAS) capable of performing the complete biochemical assay of solutions. In realizing such systems, pumps play an essential role in manipulating small, precise volumes of solution and driving them through the various components of the chip.

Existing micropumps can be broadly classified as either ‘continuous dynamic flow micropumps’ or ‘reciprocating displacement micropumps’, depending on the manner in which the working fluid is driven [1]. In micropumps of the former type, a continuous movement of the fluid is induced by means of electrohydrodynamic (EHD), electrochemical, magnetohydrodynamic (MHD), electrophoretic, electroosmotic, or impedance driving forces [2–7]. By contrast, in reciprocating displacement micropumps, the fluid is driven peristaltically by applying an oscillatory or rotational movement to a series of (typically) three stationary diaphragms [8–14]. Reciprocating micropumps are typically actuated using piezoelectric [15,16], thermopneumatic [10,11,17], pneumatic [12–14,18], electromagnetic [19], or external actuation [20,21] techniques. Seibel *et al.* [4] developed a programmable planar micropump based on the principle of electroosmotic flow (EOF). The experimental results showed that the pumping rate was bi-directionally linear and reached a maximum value of 10 nL/min at an applied voltage of 40 V. Pan *et al.* [5] presented a PDMS-membrane micropump with two one-way ball-type check valve for microfluidic applications. The micropump consisted of two functional PDMS layers, one holding the ball check valves and an actuating chamber, and the other covering the chamber and holding a miniature permanent magnet on top for actuation. An 10-turn planar coil was integrated to drive the fluid on a PC board and a pumping rate of 1 mL/min could be attained with a 500 mW of power consumption. Lee *et al.* [6,7] presented an impedance-based micropump comprising a copper microcoil, a glass microchannel, a glass cover plate and a PDMS diaphragm with a magnet mounted on its upper surface. In the proposed device, fluid was driven through the pump by passing an alternating current through the microcoil; thereby creating an electromagnetic force between the coil and the magnet and inducing a periodic deflection of the PDMS diaphragm. It was shown that a maximum diaphragm deflection of 110 μm could be obtained by supplying the coil with an actuating current of 0.6 A. Moreover, a maximum flow rate of 7.2 mL/min was achieved by driving the diaphragm at a frequency of 200 Hz. Dau *et al.* [15] proposed a MEMS-based peristaltic micropump in which the diaphragm was deflected by three piezoelectric lead zirconate titanate (PZT) actuators driven at a frequency of 7.9 kHz. The large-scale displacements of the diaphragm resulted in a significant driving pressure (280 Pa) and a substantial net flow (*i.e.*, 5.2 mL/min). However, the device required a high actuating voltage of 50 V. In 2008, Bodén *et al.* [11] presented a thermopneumatic peristaltic micropump operated by a paraffin actuator driven by a square pulse waveform. The device achieved a high head pressure of 5 MPa. However, the maximum attainable flow rate was just 1 μL/min given an actuating voltage of 1.8 V and driving frequency of 0.21 Hz. The literature contains many proposals for peristaltic micropumps actuated by cascaded pneumatic actuators [12–14,18]. Such devices are capable of generating a flow rate of 5 mL/min against zero head pressure and 2.6 mL/min against a head pressure of 25 kPa [12]. However, such flow rates are too low for many microfluidic applications, which typically require flow rates as high as 15 mL/min [6].

Of the various actuation methods available for peristaltic micropumps, electromagnetic actuation has a number of significant benefits, including an extended working range, a rapid response time and a low actuating voltage [5–7,19–22]. Accordingly, the current study develops a reciprocating pump comprising a glass lower plate containing a copper microcoil (or microcoils), a PMMA middle plate incorporating a PDMS diaphragm with a surface-mounted magnet, upper PMMA channel plates, and a ball-type check-valve located at the entrance to the PMMA channel. In the pumping operation, an AC current is passed through the microcoil; causing a bi-directional deflection of the PDMS diaphragm under the effect of the resulting electromagnetic field between the coil and the magnet. As the PDMS diaphragm oscillates in the vertical direction, the resulting change in the fluid pressure within the channel causes the check-valve to alternate continuously between the open and closed positions. Consequently, the fluid is driven periodically through the channel. The performance of the pump is evaluated experimentally for actuating currents in the range of 0∼0.5 A, actuating voltages in the range of 1.0∼3.0 V, and actuating frequencies in the range of 15∼30 Hz. It is shown that a maximum flow rate of 13.2 mL/min can be achieved using an actuating current of 0.4 A, an actuating voltage of 3 V, and a driving frequency of 15 Hz.

## 2. Micropump Design

**Figure 1.**
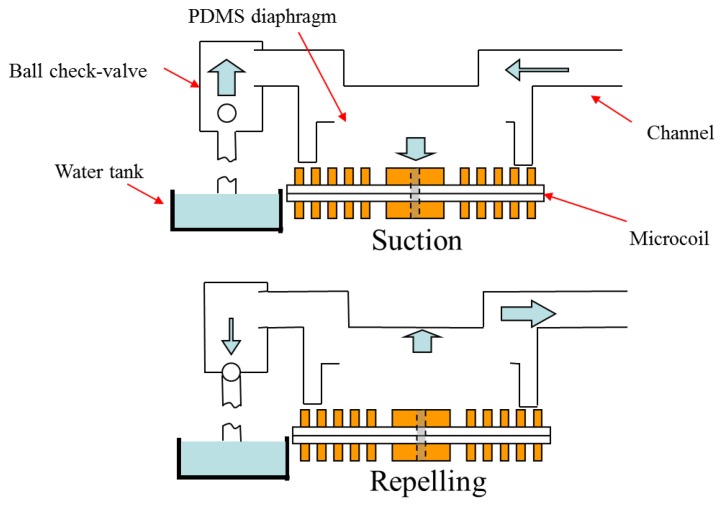
Operating principle of reciprocating pump.

Figure 1 illustrates the operating principle of the proposed pump. The pump body has overall dimensions of 50 mm × 20 mm × 18 mm (length × width × height), while the channel has a length of 13 mm and a diameter of 5 mm at the inlet and outlet, respectively. When an electrical current is passed through the microcoil, the diaphragm deflects bi-directionally, causing a periodic volume change of the channel at the frequency of the applied voltage. As the diaphragm is sucked toward the microcoil and then subsequently repelled, the resulting volume change causes the check-valve to move in the upward and downward directions, respectively. Consequently, fluid is drawn into the channel and pumped as the diaphragm deflects periodically. In practice, the flow rate is determined by both the volume change induced in the channel each time the diaphragm deflects and the frequency at which the diaphragm is actuated [23].

In designing the microcoil, the parameters of interest include the inner radius, the spacing between the individual turns, the width of each turn, and the thickness. The microcoil design procedure has two objectives, namely (1) to simplify the actuator fabrication process by minimizing the total number of coils required, and (2) to reduce the dimensions of each coil by maximizing the total number of turns [6]. Clearly, both objectives are subject to the constraint of ensuring that the microcoil stack generates a sufficient magnetic force to achieve the necessary diaphragm displacement without consuming an excessive amount of power. In the current study, actuator mechanisms comprising one, two and four microcoils were fabricated. In every case, each coil comprised 30 turns (width 150 μm and thickness 30 μm) separated by a spacing of 150 μm. Moreover, the inner radius of each coil was specified as 3.950 mm, while the outer radius was specified as 21.950 mm. The resistance of each coil (at 20 °C) was found to be 1.5 Ω.

## 3. Fabrication

**Figure 2.**
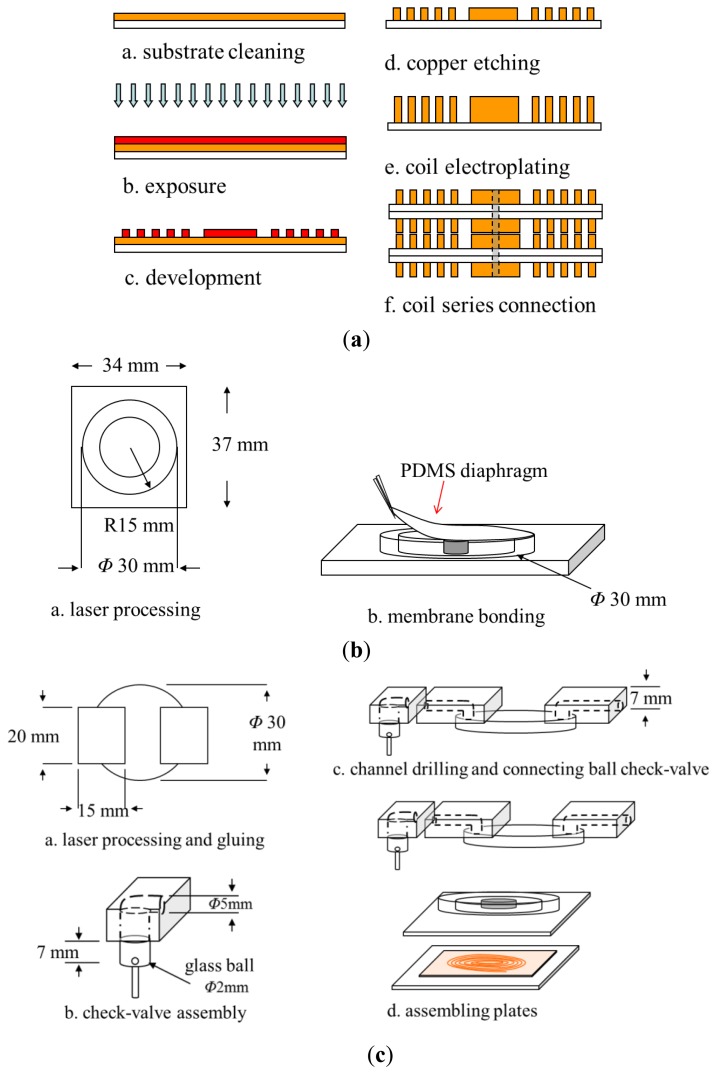
Major steps in pump fabrication process: (**a**) microcoil patterning and deposition, (**b**) PDMS diaphragm attachment to PMMA middle plate, and (**c**) pump assembly.

The proposed pump was fabricated using conventional photolithography, electroplating and wet etching micro-fabrication techniques. As shown in Figure 2, the fabrication process involved the following basic procedures: (1) electroplating the microcoil on the lower substrate; (2) laser engraving the channel configuration on the middle PMMA substrate; (3) fabricating the PDMS diaphragm; (4) mounting the permanent magnet on the lower surface of the diaphragm; (5) attaching the diaphragm to the middle PMMA substrate; (6) drilling via holes of diameter 5 mm in the upper PMMA substrates to form the inlet and outlet of the pump, respectively; (7) connecting a ball check-valve to the inlet of the upper PMMA substrates; and (8) bonding the upper, middle and lower substrates to form a sealed pump. The step-by-step procedures used in fabricating the microcoil and pump are described in the sections below.

### 3.1. Microcoil Stack

Figure 2(a) illustrates the major steps in the microcoil stack fabrication process. Briefly, the procedure can be described as follows:

1. A copper clad laminate (CCL) substrate comprising an upper copper layer (18 μm) and a lower polyimide layer (18 μm) was cleaned in acetone solution.
2. A photoresist layer (AZ 4620, Clariant Corp., Muttenz, Switzerland) with a thickness of 25 μm was deposited on the copper surface using a spin coating technique.
3. The PR layer was patterned with the desired microcoil geometry using a photolithography technique.
4. The copper not covered by the patterned PR layer was etched and the residual PR was then stripped away in acetone solution.
5. A copper layer with a thickness of 12 μm was deposited in the microcoil mold by means of an electroplating process.
6. Two or more microcoils were series connected in a vertical direction by welding through via holes drilled in the coil center.

**Figure 3.**
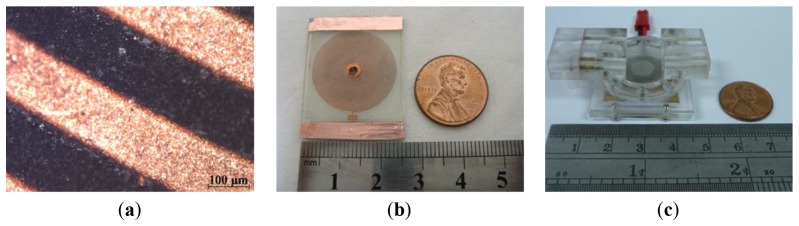
Photographs of: (**a**) microcoil surface, (**b**) microcoil on lower glass plate, and (**c**) completed pump.

Figure 3(a,b) presents photographs of the microcoil surface and the lower glass substrate containing the microcoil, respectively.

### 3.2. Micropump

Finally, the upper microchannel layer, middle magnet/diaphragm layer and lower microcoil layer were bonded using UV glue to form the finished micropump (see Figure 2(c)). Figure 3(c) presents a photograph of the completed device.

## 4. Results and Discussion

**Figure 4.**
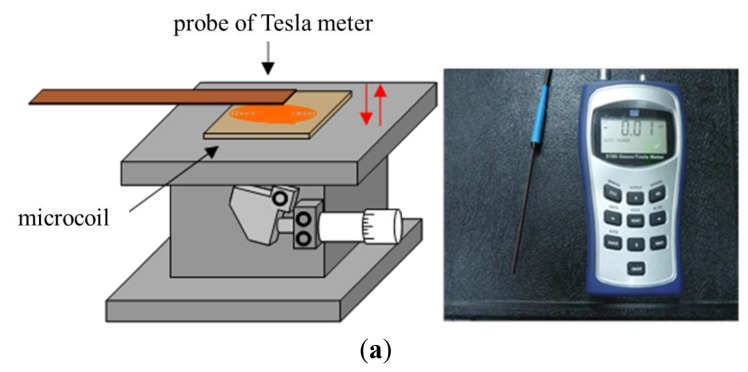

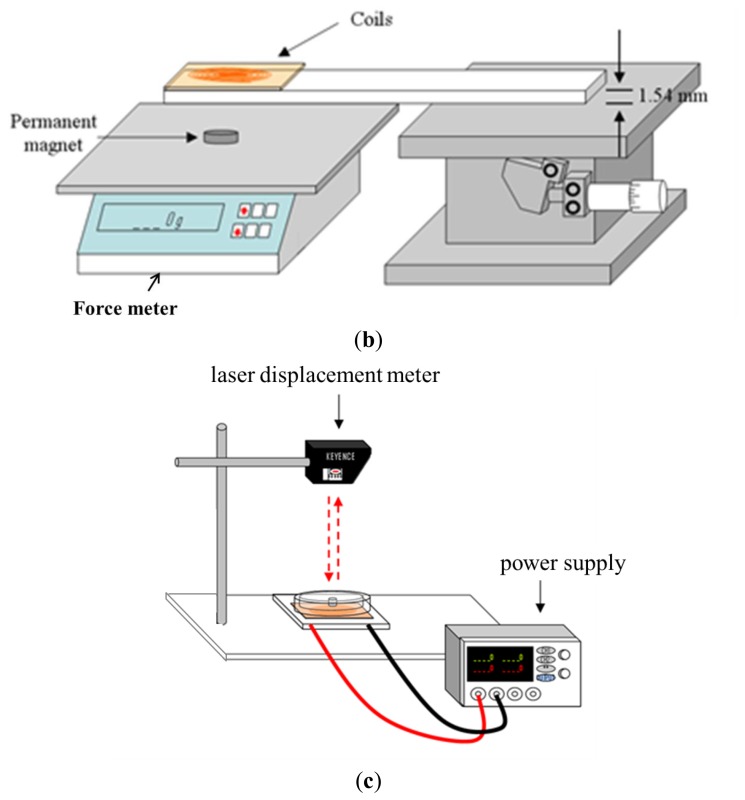
Measurement instrument set-ups of (**a**) flux density, (**b**) electromagnetic force and (**c**) membrane displacement.

**Figure 5.**
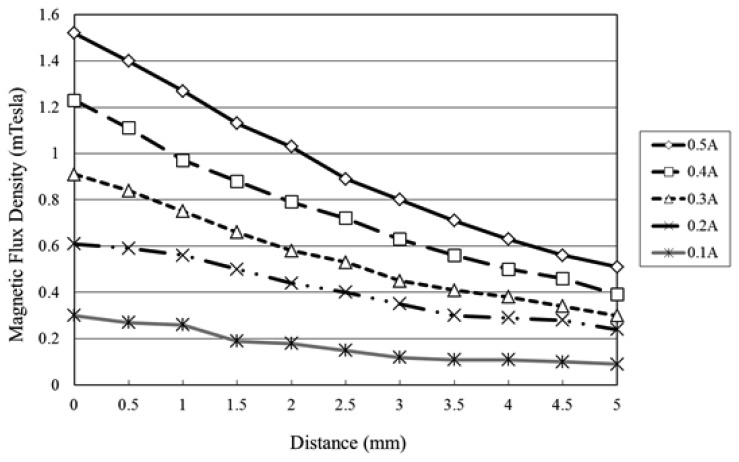
Variation of magnetic flux density produced by single-layer microcoil stack with vertical distance from microcoil surface given input currents of 0∼0.5 A.

**Figure 6.**
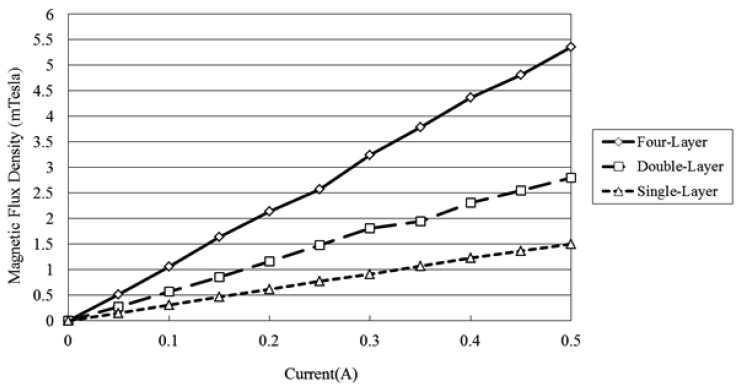
Variation of magnetic flux density produced at center of single-, double- and four-layer microcoil stacks given input currents of 0∼0.5 A.

The flux density characteristics of the microcoil stack were measured using a Tesla meter (TM-401, KANETEC, Nagano-ken, Japan; Figure 4(a)). In the tests, the stack was supplied with an input current of 0∼0.5 A and the variation of the flux density was measured in the vertical direction along the central axis of the coil. The corresponding results are presented in Figure 5 (note that the results correspond to a single microcoil). It can be seen that for a constant coil current, the magnetic flux density decreases with an increasing distance from the coil surface. Moreover, for a constant distance, the magnetic flux intensity increases with an increasing current. From inspection, the maximum magnetic flux density is found to be 1.53 mTesla (obtained at the center of the coil surface given a coil current of 0.5 A). Figure 6 presents the variation of the magnetic flux density with the coil current (0∼0.5 A) given microcoil stacks with one, two and four layers (coils), respectively. As expected, the magnetic flux density increases with both an increasing coil current and an increasing number of coils due to stronger magnetic fields. The maximum magnetic flux density is found to be 5.40 mTesla given a coil current of 0.5 A and a stack comprising four layers.

**Figure 7.**
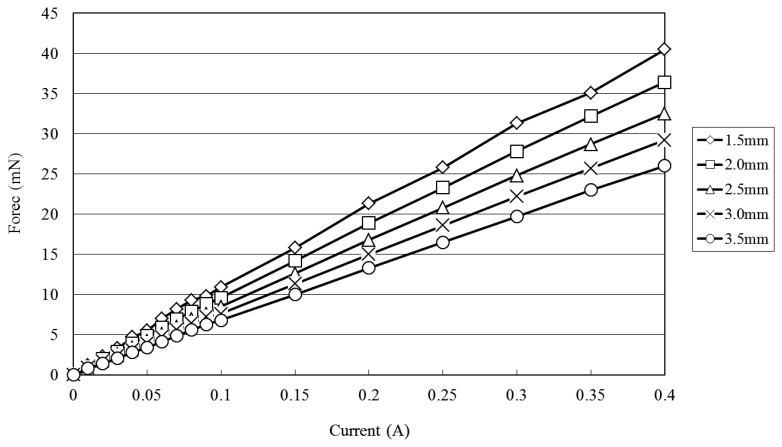
Variation of electromagnetic force with input current (0∼0.4 A) at various vertical distances from microcoil surface (Note that results correspond to single-layer microcoil stack).

**Figure 8.**
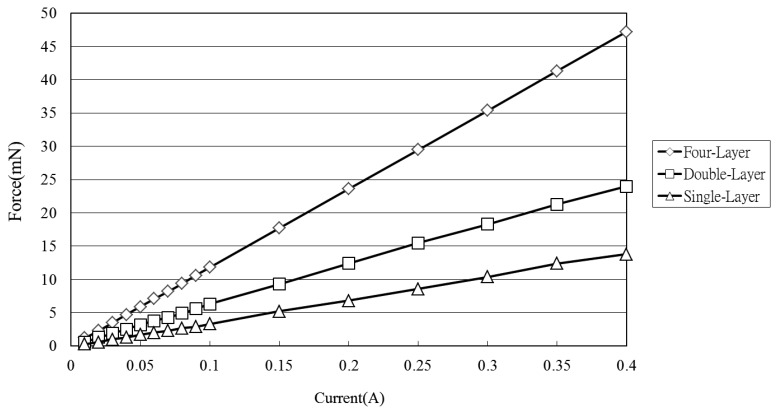
Variation of electromagnetic force with input current (0∼0.4 A) at vertical distance of 1.5 mm from surface of single-, double- and four-layer microcoil stacks.

Figure 7 shows the variation of the electromagnetic force developed at the center of the diaphragm with the coil current (0∼0.4 V) given various coil-to-diaphragm separation distances. (Note that the results were obtained using a Sky-300 force meter (MITSUMASA, Kaohsiung, Taiwan; Figure 4(b)) and correspond to a stack with a single microcoil.) It is seen that the electromagnetic force increases due to the increased magnetic flux caused by the increased electric current and the decreased vertical distance from the coil center and the electromagnetic force exceeds 40 mN at a vertical distance of 1.5 mm given an input current of 0.4 A. Figure 8 shows the electromagnetic force developed at a distance of 1.5 mm from the coil center given input currents in the range of 0∼0.4 A and microcoil stacks comprising one, two and four microcoils, respectively. As expected, the electromagnetic force increases with an increasing number of microcoils. The maximum electromagnetic force is found to be 47 mN given an input current of 0.4 A and a four-layer microcoil stack.

**Figure 9.**
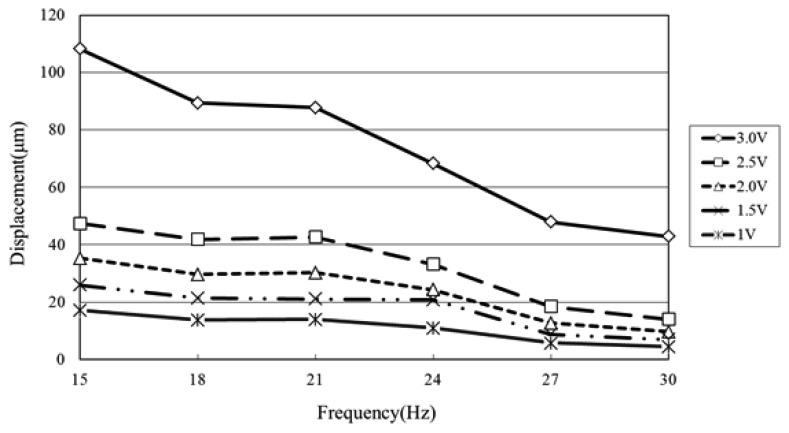
Variation of maximum diaphragm deflection with excitation frequency (15∼30 Hz) given actuating voltages ranging from 1.0∼3.0 V. (Note that results correspond to four-layer microcoil stack.)

The displacement characteristics of the diaphragm given actuating voltages in the range of 15∼30 Hz were measured using a laser displacement meter (LC-2400A + 2430, Keyence, Osaka, Japan) powered by a PR8323 power supply (ABM, Hsinchu, Taiwan; Figure 4(c)). The displacement was measured at the center point of the diaphragm (*i.e.*, the position of maximum deflection) for microcoil voltages in the range of 1.0∼3.0 V. The corresponding results are presented in Figure 9 (Note that the results correspond to a four-layer microcoil). It is seen that the diaphragm displacement increases with an increasing driving voltage due to the greater electromagnetic force. However, the displacement reduces as the driving frequency increases due to the response time limit of the diaphragm movement. From inspection, the maximum displacement is found to be 108 μm given an applied voltage of 3.0 V and driving frequency of 15 Hz. It can be found the displacement changes much higher as the applied voltage is 3.0 V because the displacement is over the elastic limit of the membrane.

**Figure 10.**
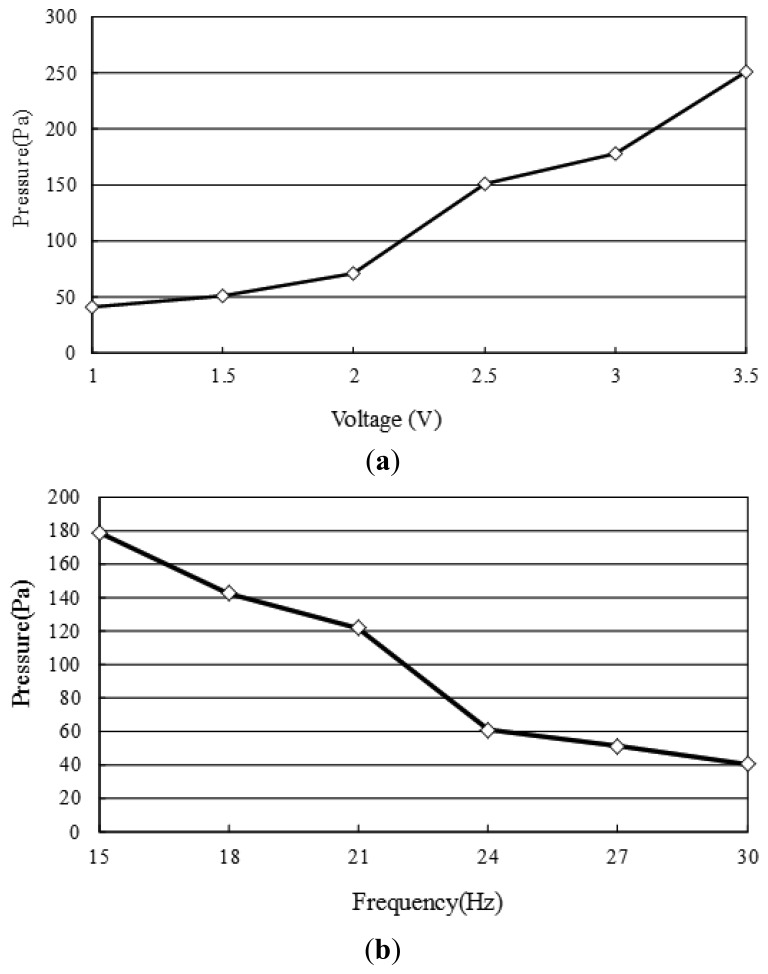
Variation of head pressure given: (**a**) applied voltage ranging from 1∼3.5 V and excitation frequency of 15 Hz, and (**b**) excitation frequency ranging from 15∼30 Hz and applied voltage of 3.0 V.

**Figure 11.**
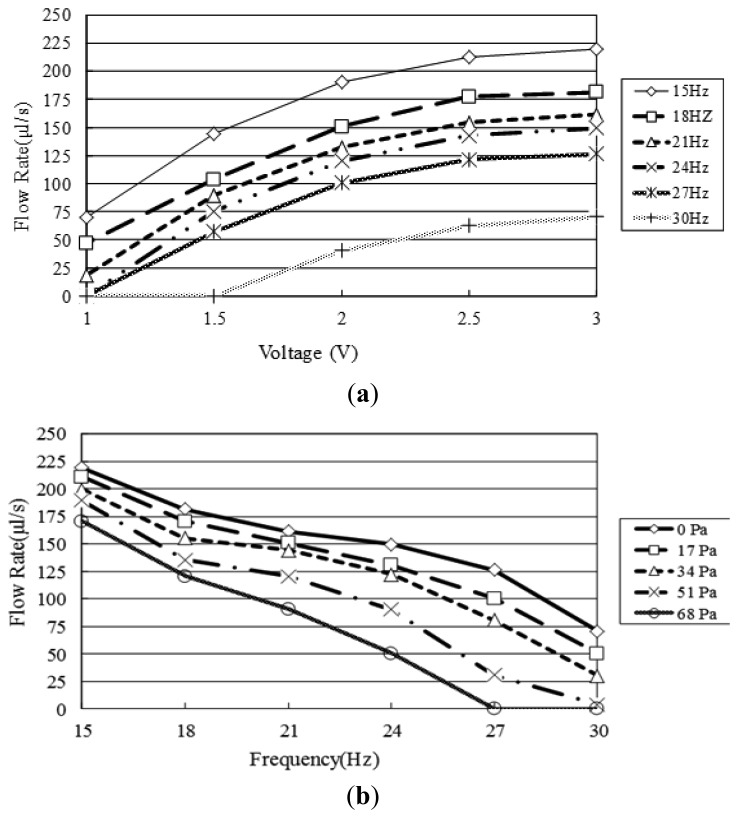
Variation of flow rate with actuation frequency (15∼30 Hz) given: (**a**) applied voltage ranging from 1.0∼3.0 V, and (**b**) head pressure ranging from 0∼68 Pa.

Figures 10 and 11 show the pressure build-up and flow rate characteristics of the four-coil pump given different values of the actuating voltage and frequency, respectively. Note that the pressure build-up was measured by monitoring the vertical distance of the inlet of the pump and the water tank (Figure 1). Meanwhile, the flow rate was measured by monitoring the reduction in the water level on the input side. Figure 10 shows that the head pressure increases with an increasing actuating voltage due to a greater electromagnetic force, but decreases with an increasing frequency due to a slower movement response, respectively. Figure 10(a) shows that the pump develops a maximum pressure of 250 Pa given an applied voltage of 3.5 V and driving frequency of 15 Hz. Figure 11(a) shows the variation of the flow rate with the actuating voltage given driving frequencies ranging from 15∼30 Hz and a zero head pressure. It is seen that a maximum flow rate of 220 μL/sec (13.2 mL/min) is obtained given an applied voltage of 3 V and driving frequency of 15 Hz. Figure 11(b) shows the variation of the flow rate with the excitation frequency given head pressures ranging from 0∼68 Pa. As expected, the flow rate decreases with an increasing head pressure at all values of the driving frequency. For a head pressure of 51 Pa or more, the flow rate reduces to zero given driving frequencies higher than 27 Hz. Please see the video of the proposed pump integrated with a microfluidic chip to drive polymer microspheres (diameter: 10 μm) in it. In the current experiments, the temperature rises of the microcoils and the driven fluid are 6.7 °C and 0.2 °C, respectively. It is observed that the former one is low enough not to affect the dynamic behavior of the electromagnetic actuator and the latter one is extremely small due to the thick thermal isolation of the bottom of the middle substrate. In the experiments of the study, it can be found that the optimal operation frequency of the proposed pump is 15 Hz (which is much lower that its response frequency—34.7 kHz [24]).

## 5. Conclusions

This study has designed, fabricated and characterized a novel reciprocating pump comprising a microcoil stack, a PDMS diaphragm with a surface mounted permanent magnet, a PMMA channel plate, and a ball-type check valve. The experimental results have shown that the actuator mechanism provides a large diaphragm deflection, a high magnetic field energy density, and a low power consumption. It has been shown that a maximum diaphragm deflection of 108 μm can be obtained using a four-layer microcoil stack, an actuating current of 0.4 A and a driving frequency of 15 Hz. The corresponding flow rate is equal to 13.2 mL/min. The pump is fabricated using conventional MEMS techniques and has a planar structure and can therefore be readily integrated with other microfluidic devices to create a Lab-on-a-Chip device (e.g., drug delivery, droplet formation, sample and reagent driving, *etc*.). Overall, the experimental results indicate that the pump presented in this study provides an easy concept of pump assembly and an ideal solution for microfluidic systems in which a relatively high pumping rate is required.
